# Impact of Adipokine Expression on Tumor Characteristics and Survival Outcomes in Patients with Renal Cell Carcinoma

**DOI:** 10.3390/medicina61091544

**Published:** 2025-08-27

**Authors:** Oktay Halit Aktepe, Tugce Ulasli, Aytac Terzi, Fatma Gundogdu, Eda Caliskan Yildirim, Ahmet Melih Arslan, Huseyin Salih Semiz, Kemal Kosemehmetoglu, Aziz Karaoglu, Mustafa Erman, Suayib Yalcin

**Affiliations:** 1 Department of Medical Oncology, Dokuz Eylul University, Izmir 35330, Turkey; tugce.ulaslialtun@deu.edu.tr (T.U.);; 2 Department of Pathology, Hacettepe University, Ankara 06230, Turkey; 3 Department of Medical Oncology, Sincan Research and Training Hospital, Ankara 06949, Turkey; 4 Department of Medical Oncology, Hacettepe University Cancer Institute, Ankara 06230, Turkey

**Keywords:** adipokine, adiponectin, leptin, renal cell carcinoma, resistin

## Abstract

*Background and Objectives*: To investigate the clinical significance of adipokines’ [leptin, leptin receptor (leptin-R), adiponectin, and resistin] expression on the characteristics and survival outcomes of patients with renal cell carcinoma (RCC). *Materials and Methods*: A total of 81 patients were included. The expressions of adipokines in the nephrectomy material of the patients were assessed using immunohistochemistry. Staining patterns were divided into two groups for statistical analyses: negative (no staining) and positive. Univariable and multivariable Cox regression models were used to evaluate the impact of the expression of adipokines on the survival outcomes of the patients. *Results*: The percentages of patients expressing leptin, leptin-R, adiponectin, and resistin were 36.4%, 30.7%, 32%, and 60.2%, respectively. The median overall survival (OS) of all patients was 53.7 months (95% confidence interval [CI]: 39.9–67.5). In the multivariate analyses, only leptin expression status was associated with OS among adipokines (hazard ratio [HR]: 1.98, 95%CI: 1.03–3.78, *p* = 0.039) in addition to the presence of distant metastasis (HR: 2.48, 95%CI: 1.16–5.29, *p* = 0.018). No significant associations were determined between adipokine expression and pathologic determinants of RCC, including tumor stage, grade, and histological subtype. *Conclusions*: Our study demonstrated that leptin expression was an independent prognostic factor for inferior OS in RCC patients treated with nephrectomy, even after adjusting for disease stage in multivariate analysis.

## 1. Introduction

Kidney cancer is the 12th most commonly diagnosed cancer worldwide, accounting for approximately 2.0% of all new cancer cases [1]. Various risk factors have been identified for renal cell carcinoma (RCC), including a hereditary history of kidney cancer, hypertension, smoking, chronic kidney disease, and obesity [2]. In the past decades, due to the increasing rate of obesity, the relationship between obesity and RCC development has gained attention in comparison with other risk factors. A meta-analysis study including 21 cohort trials demonstrated that obesity [body mass index (BMI) ≥ 30.00 kg/m^2^] and pre-obese status (BMI: 25.00–29.99) were highly associated with RCC development risk compared to people of standard weight [hazard ratio (HR): 1.77, 95% confidence interval (CI): 1.68–1.8; HR: 1.28, 95% CI: 1.24–1.33, respectively] [3].

Adipocyte hyperplasia, hypertrophy, and increased intracellular lipids are the hallmarks of adipose tissue enlargement, which is the main manifestation of obesity [4]. Adipose tissue secretes adipokines, namely leptin, adiponectin, and resistin, which play a crucial role in the regulation of tissue homeostasis. When this regulation does not function properly, it results in an obese state through metabolic imbalance [5]. Since RCC is one of the well-known obesity-related cancers, adipokines have gained immense importance in terms of risk estimation and prognosis. Preoperative serum leptin and overexpression of the leptin receptor (leptin-R) were linked to tumor invasiveness and progression of RCC [6]. It has been reported that patients with lower adiponectin levels exhibit aggressive pathological features and poorer survival outcomes than those with higher levels of adiponectin in RCC patients undergoing nephrectomy [7]. Furthermore, a study by Kallio et al. demonstrated that elevated resistin levels are associated with advanced disease status and poorer survival outcomes in RCC [8]. Previous studies on adipokines in RCC have primarily focused on their circulating levels in the blood [6,7,8]. These studies explored associations between serum adipokine concentrations and both survival outcomes and tumor characteristics. Thus, to the best of our knowledge, in this study, we report for the first time the predictive value of the immunohistochemical (IHC) status of leptin, leptin-R, adiponectin, and resistin together on tumor characteristics and outcomes in a cohort of 81 RCC patients who were treated with nephrectomy.

## 2. Materials and Methods

### 2.1. Patient Population

RCC patients who were ≥18 years and treated with radical or partial nephrectomy between 2008 and 2019 at Hacettepe University Cancer Institute were included. Patients who received neoadjuvant or adjuvant treatment before nephrectomy were excluded. The clinicopathological characteristics, including age, sex, tumor grade, and lymphovascular invasion (LVI), were collected using pathology reports and medical records of patients from our center’s electronic system. All histologic subtypes were evaluated according to the 8th edition of the AJCC TNM staging system [9]. In our center, Fuhrman and Paner systems have been used to grade clear-papillary and chromophobe RCCs, respectively [10,11]. Patient risk stratification at the metastatic setting was evaluated in the International Metastatic Renal Cell Carcinoma Database Consortium (IMDC) system, and patients were categorized into three risk groups: favorable, intermediate, and poor [12]. The study was approved by the Clinical Research Ethic Commission of Hacettepe University and performed according to the latest version of the Declaration of Helsinki.

### 2.2. IHC

To prepare tissue microarrays (TMA), hematoxylin and eosin (H&E) stained preparations belonging to each patient were examined first. Appropriate tumor areas were selected for the TMA. From the selected areas of RCC tissue blocks, tumor cores with a diameter of 3 mm were extracted using a manual punch arrayer and transferred into a newly constructed recipient paraffin block designed to contain 40 tissue cores. Multiple sections with a thickness of 4 μm were taken from the TMA blocks. The first of these sections was stained with H&E and examined with light microscopy to confirm the presence of representative tumor areas. IHC studies were performed using the Bond Polymer Dense Detection System (Leica Microsystems, Victoria, Australia) in accordance with the instructions. The 4 μm thick sections of formalin-fixed and paraffin-embedded TMA tissues were deparaffinized in Bond Devax solution (Leica Microsystems) at 72 degrees. A mouse monoclonal anti-adiponectin antibody (ab22554, 1:100; Abcam, Cambridge, UK) and a mouse monoclonal resistin antibody [(C-10): sc-376336, 1:100, Santa Cruz Biotechnology, Inc., Dallas, TX, USA] were incubated in ER1 for 20 min, a mouse monoclonal anti-leptin-R antibody [(B-3): sc8391, 1:200, Santa Cruz Biotechnology, Inc., Dallas, USA] and a mouse monoclonal anti-leptin antibody [(F-3), sc48408, 1:200, Santa Cruz Biotechnology, Inc., Dallas, USA] were incubated in ER2 for 10 min at 100 degrees for pre-treatment. The incubation time for the primary antibody was 40 min. A blockade with hydrogen peroxide was carried out for 10 min. Tissues treated with post-primer for 7 min, with polymer for 7 min, and with DAB for 7 min were passed through alcohol and xylene and closed. The IHC expressions of the adipokines were scored as negative (no tumor cells were reactive) and positive (≥1% tumor cells were reactive) for statistical analyses.

### 2.3. Statistical Analysis

Differences in continuous and categorical parameters were evaluated using independent samples t-test and chi-square test, respectively. The overall survival (OS) of the whole cohort was measured from diagnosis to death from any cause or the last follow-up. Multivariate Cox analyses were performed to evaluate the associations between survival outcomes and clinicopathological variables, including those with statistical significance or potential prognostic value, based on prior literature or univariate analysis. Regarding patients treated with targeted therapy, progression-free survival (PFS) was defined as the time interval between the date of treatment initiation and the date of tumor progression and/or death and OS was defined as the time length from treatment initiation to the last follow-up and/or death from any cause. All statistical analyses were conducted with IBM SPSS Statistics, version 25.0 (IBM Corp., Armonk, NY, USA) software, and a type-I error level of 5% (*p* < 0.05) was considered for statistical significance.

## 3. Results

### 3.1. Baseline Patient Characteristics and Study Design

Baseline patients’ characteristics of the whole cohort and the groups that were determined according to leptin expression status are presented in Table 1. A total of 81 patients who underwent nephrectomy were enrolled. The median age was 61 (range: 29–81), and 70.3% of the patients were male. At initial diagnosis, 48.1% of the patients had distant metastasis, whereas 22.2% of the patients had lymph node metastasis. Additionally, our cohort consisted of mostly T3–4 tumors (90.1%). The significant proportion of the cohort was composed of patients with clear cell histology and tumor grade III-IV (82.7% and 79%, respectively). The presence of LVI was found in 53% of the patients.

### 3.2. Adipokine Expression and Their Association with Pathological Variables and Survival Outcomes in RCC

**Figure 1:**
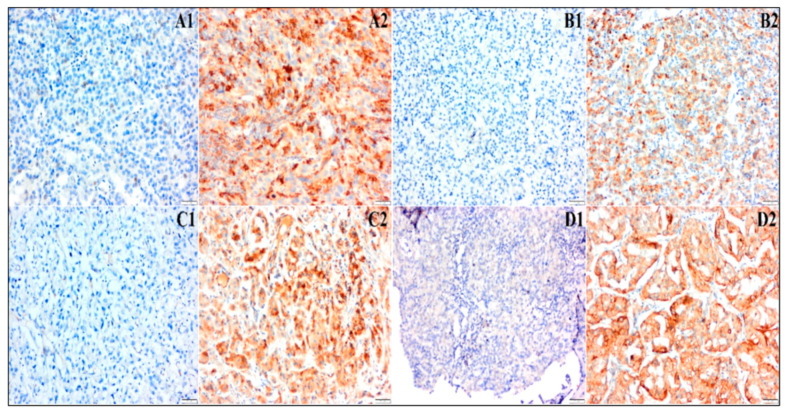
Representative images of negative and positive expressions of leptin ((**A1**) negative, (**A2**) positive), leptin receptor ((**B1**) negative, (**B2**) positive), adiponectin ((**C1**) negative, (**C2**) positive), and resistin ((**D1**) negative, (**D2**) positive).

Leptin and leptin-R expression was detected in 36.4% and 30.7% of the patients, respectively. However, 32% and 60.2% of the patients had adiponectin and resistin expression, respectively. Negative and positive expressions of adipokines are presented in Figure 1. Spearman correlation test was used to determine relationships between adipokines, whereby leptin expression was significantly correlated with leptin-R (*r* = 0.362, *p* = 0.001) and resistin expression (*r* = 0.279, *p* = 0.013). None of the expressions of adipokines were associated with any pathological characteristics of RCC.

**Figure 2:**
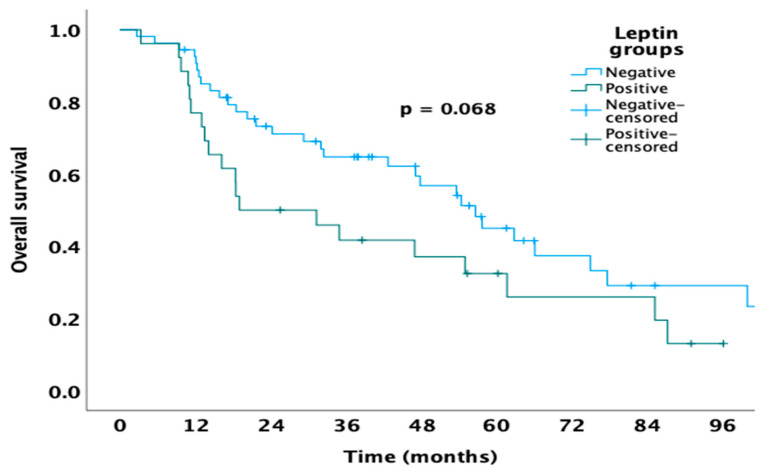
Kaplan–Meier analyses estimating OS of patients treated with nephrectomy stratified according to leptin expression status.

During the median follow-up time of 37.4 months (minimum–maximum: 2.6–163.7), 51 patients died. The median OS in the cohort was 53.7 months (95% CI: 39.9 to 67.5). Although not statistically significant, patients without leptin expression showed a trend toward a longer median OS compared to those with detectable expression (56.7 vs. 19 months, *p* = 0.068, respectively) (Figure 2).

**Figure 3:**
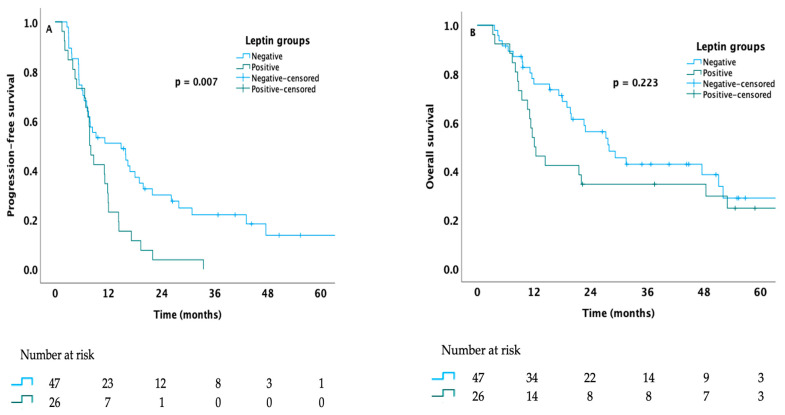
Kaplan–Meier curves estimating the PFS (**A**) and OS (**B**) of metastatic RCC patients treated with targeted therapy stratified according to leptin expression status.

Regarding patients who were treated with targeted therapy, pazopanib, or sunitinib, the median PFS of patients with no leptin expression was significantly higher than that of patients with detectable leptin expression (14.9 months vs. 7.8 months, *p* = 0.007, respectively), whereas the median OS time of patients with no leptin expression was longer but not significantly different than those with leptin expression (27.9 months for no leptin expression group vs. 12.1 months for leptin expression group, *p* = 0.223) (Figure 3).

As presented in Table 2, the univariate analyses demonstrated that the clinical variables associated significantly with poorer OS were age ≥65 (HR: 1.99, 95% CI: 1.09–3.61, *p* = 0.023), the presence of nodal status (HR: 2.52, 95% CI: 1.36–4.65, *p* = 0.003), and the presence of distant metastasis (HR: 2.79, 95% CI: 1.56–5.02, *p* = 0.001). The potential variables in univariate analyses for prediction of OS were histology (HR: 0.54, 95% CI: 0.22–1.29, *p* = 0.170), tumor grade (HR: 1.93, 95% CI: 0.90–4.12, *p* = 0.09), LVI (HR: 1.75, 95% CI: 0.97–3.15, *p* = 0.063), and the presence of leptin expression (HR: 1.69, 95% CI: 0.95–2.99, *p* = 0.071). However, applying multivariate analyses with significant and potential variables, we demonstrated that the independent factors in predicting OS were leptin expression (HR: 1.98, 95% CI:1.03–3.78, *p* = 0.039) and the presence of distant metastasis (HR: 2.48, 95% CI: 1.16–5.29, *p* = 0.018) (Table 2). Considering the associations of leptin-R, adiponectin, and resistin expression levels with OS, no significant relationship was found.

## 4. Discussion

The primary hypothesis of this study is that the IHC expression of adipokines—specifically leptin, leptin-R, adiponectin, and resistin—in RCC is associated with distinct tumor characteristics and may independently predict OS in patients undergoing nephrectomy. Our findings strongly suggest that only leptin expression among adipokines is an independent factor in predicting OS in RCC patients undergoing nephrectomy.

Adipokines are bioactive molecules secreted by adipose tissue that can regulate RCC tumor biology through various mechanisms. Leptin promotes tumor growth by activating the JAK/STAT and PI3K/AKT signaling pathways, leading to increased proliferation, angiogenesis, and inhibition of apoptosis [13]. Leptin can also increase tumor-associated inflammation by upregulating pro-inflammatory cytokines [14]. In contrast, adiponectin is generally considered anti-tumorigenic and has effects such as suppressing proliferation and inducing apoptosis, which are likely mediated by the AMP-activated protein kinase (AMPK) and peroxisome proliferator-activated receptor (PPAR)-α pathways [15]. Resistin plays a role in cancer progression by activating the nuclear factor kappa B (NF-κB) signaling pathway and promoting inflammatory responses [16]. These mechanical perspectives suggest that altered adipokine expression in RCC may contribute to tumor aggressiveness and affect patient outcomes.

Leptin, a peptide hormone produced by adipocytes, was first described in 1994, and a strong association was determined between leptin and homeostasis of body weight regulation [17]. Leptin level is directly correlated with the amount of adipose tissue, so obese people have increased levels of leptin in comparison to non-obese people [18]. Leptin exhibits its function via leptin-R, a membrane-spanning receptor, which is categorized in the class I cytokine receptor family [19]. Leptin may regulate the carcinogenesis process by downregulating apoptosis [20], increasing the production of macrophage-derived inflammatory cytokines [21], and promoting angiogenesis [14]. Regarding the impact of leptin on clinicopathological parameters and survival outcomes of RCC patients, Horiguchi et al. reported that a high leptin expression status is associated with regional lymph node metastasis (*p* = 0.047), the presence of venous invasion (*p* = 0.013), and histological type (*p* = 0.0076) [6]. In the same study, while a high serum leptin level was demonstrated as an independent factor for PFS (*p* = 0.0406), the associations between leptin-R expression status and cancer-related survival and PFS times were not significantly different [6]. Choi et al. prospectively evaluated the associations between the plasma levels of four plasma adipokines, leptin, adiponectin, retinol binding protein 4 (RBP-4), nicotinamide phosphoribosyltransferase (NAMPT), and RCC, and they determined that adiponectin, RBP-4, and NAMPT, but not leptin, were significantly associated with RCC [22]. Furthermore, a meta-analysis aimed to explore the association between serum leptin level and RCC susceptibility and progression and showed that leptin level was not related to the progression of RCC [23]. A Mendelian randomization study also demonstrated that circulating leptin was not associated with the risk of obesity-related cancers, including RCC [24]. A previous study by Perumal et al. investigated leptin and leptin-R expression in clear cell RCC using IHC and reported no significant association with survival outcomes, except for nuclear leptin expression in Kaplan–Meier analysis [25]. Our study expands on this by evaluating the combined IHC expression of leptin, leptin-R, adiponectin, and resistin in a broader RCC cohort encompassing all histologic subtypes, and by identifying leptin expression as an independent prognostic factor for OS in multivariate analysis. Additionally, in this study, only 20% of their cohort initially had metastatic disease, and they did not show follow-up time and patients’ treatment types received in the metastatic setting [25]. In the present study, almost half of our cohort (48.1%) was composed of initially metastatic RCC patients, and the median follow-up time was 37.4 months. Regarding survival analyses of the patients, detectable leptin expression and presence of distant metastasis at initial diagnosis were found to be independent determinants for OS.

Regarding the association between leptin-R and survival outcomes of RCC, Lee et al. investigated the impact of leptin-R expression on clinicopathologic characteristics and survival of 110 upper tract urothelial carcinomas treated with nephroureterectomy and excision of the bladder cuff, and they showed that patients with higher leptin-R expression (>50%) had poorer CSS (HR: 2.26; *p* = 0.006) and RFS (HR: 2.55; *p* = 0.011) [26]. Taking leptin-R into consideration, its expression has been identified at both the mRNA and protein levels in RCC cell lines, including the murine Renca kidney cancer cell line [27]. In addition to leptin, leptin-R expression has been determined in diversified cancer types, including RCC [28,29,30]. Based on these findings, the leptin/leptin-R signal pathway could be linked to the pathogenesis of several tumors, especially RCC, in which obesity plays an important role as a causative risk factor [31].

Adiponectin, a polypeptide containing 244 amino acids, is mainly derived and secreted from white adipose tissue [32]. Considering the functional roles of adiponectin, its association with obesity is of prime importance because obese subjects have paradoxically lower serum adiponectin concentration than non-obese ones [32]. Several reports have demonstrated that people who have relatively reduced concentration levels of serum adiponectin are found to carry an increased risk for obesity-associated cancers, including RCC [33,34,35]. However, in the present study, no relationship between adiponectin expression and the outcomes of RCC patients was investigated.

Resistin is a cysteine-rich protein that is secreted primarily from macrophages found in adipose tissue [36]. In addition to leptin and adiponectin, it is linked to insulin resistance, inflammation, and adipogenesis [37]. Liao et al. showed that circulating resistin level was not associated with clear cell RCC risk [38]. However, a recent report by Zhang et al. demonstrated that resistin mRNA expression in clear cell RCC was significantly higher than in tumor-adjacent normal tissue [39]. To the best of our knowledge, this is the first study to evaluate the association between resistin expression and both tumor characteristics and OS in RCC. Our results exhibited that resistin expression was observed in 60.2% of the tumors, and no significant relationship was recorded between resistin and any pathologic features and OS of RCC.

Nevertheless, some limitations of the present study should be noted. Firstly, the present study has a retrospective nature, and it is composed of mostly T3-T4 and tumor grade III-IV tumors because we only included the patients who were treated and followed in our clinic. Secondly, since TMA was used, we could not determine the expression of adipokines in normal kidney tissue adjacent to the tumor. Thirdly, we could not analyze adipokine expression and serum adipokine levels simultaneously. Fourthly, metastatic RCC patients in our cohort were treated with targeted agents in contrast to the current combination treatment with immunotherapy and targeted agents.

## 5. Conclusions

The present study provides important evidence about the association between leptin expression and OS of RCC patients undergoing nephrectomy. Further prospective and in vitro experiments are needed to explore the exact role of adipokines in the pathogenesis and prognosis of RCC.

## Figures and Tables

**Table 1:** Baseline clinicopathologic characteristics of the patients stratified based on leptin expression status.

Characteristics	All Patients (*n* = 81)	No Leptin Expression (*n* = 54)	Leptin Expression (*n* = 27)	*p* Value
Age (years), range	61 (29–81)	61 (29–81)	61 (30–77)	0.750
Gender				0.592
Female	24 (29.7%)	15 (27.8%)	9 (33.3%)	
Male	57 (70.3%)	39 (72.2%)	18 (66.7%)	
BMI (kg/m^2^)	26.1 (24.2–30)	26.2 (24.2–30.3)	25.7 (24.2–29.7)	0.597
Nephrectomy type				0.522
Radical	69 (85.2%)	47 (87%)	22 (81.5%)	
Partial	12 (14.8%)	7 (13%)	5 (18.5%)	
Histology				0.317
Clear cell	67 (82.7%)	44 (81.5%)	23 (85.1%)	
Papillary	6 (7.4%)	3 (5.5%)	3 (11.1%)	
Chromophobe	8 (9.9%)	7 (13%)	1 (3.8%)	
Presence of LVI				0.081
Yes	43 (53%)	25 (46.2%)	18 (66.7%)	
No	38 (47%)	29 (53.8%)	9 (33.3%)	
T stage				0.795
I–II	8 (9.9%)	4 (7.4%)	4 (14.8%)	
III–IV	73 (90.1%)	50 (92.6%)	23 (85.2%)	
Presence of lymph node metastasis				0.133
Yes	18 (22.2%)	9 (16.6%)	9 (33.3%)	
No	63 (77.8%)	45 (83.4%)	18 (66.7%)	
Presence of distant metastasis				0.941
Yes	39 (48.1%)	26 (48.1%)	13 (48.1%)	
No	42 (51.9%)	28 (51.9%)	14 (51.9%)	
Tumor grade				0.233
I–II	17 (21%)	11 (20.3%)	6 (22.2%)	
III–IV	64 (79%)	43 (79.7%)	21 (77.8%)	

Continuous variables are presented as median with interquartile range. Categorical variables were presented as frequencies and percentages. Abbreviations: BMI: body mass index; LVI: lymphovascular invasion.

**Table 2:** Associations of clinicopathologic variables with OS of RCC.

	Univariate		Multivariate	
Variable	HR (95% CI)	*p* Value	HR (95% CI)	*p* Value
Age (<65 versus ≥65)	1.99 (1.09–3.61)	0.023	1.87 (0.85–4.10)	0.116
Gender (Female vs. Male)	1.06 (0.59–1.91)	0.839	N/A	N/A
T stage (I–II vs. III–IV)	3.42 (0.46–25.13)	0.226	N/A	N/A
Lymph node status (N-negative vs. N-positive)	2.52 (1.36–4.65)	0.003	1.81 (0.80–4.09)	0.150
Metastasis status (M-negative vs. M-positive)	2.79 (1.56–5.02)	0.001	2.48 (1.16–5.29)	0.018
Histology (Clear cell vs. Non-clear cell)	0.54 (0.22–1.29)	0.170	0.53 (0.17–1.59)	0.262
Tumor grade (I–II vs. III–IV)	1.93 (0.90–4.12)	0.090	1.38 (0.55–3.49)	0.487
LVI status (Absent vs. Present)	1.75 (0.97–3.15)	0.063	0.97 (0.45–2.08)	0.957
Leptin expression (Absent vs. Present)	1.69 (0.95–2.99)	0.071	1.98 (1.03–3.78)	0.039
Leptin-R expression (Absent vs. Present)	1 (0.56–1.77)	0.988	N/A	N/A
Adiponectin expression (Absent vs. Present)	1.17 (0.65–2.13)	0.590	N/A	N/A
Resistin expression (Absent vs. Present)	1.114 (0.62–2)	0.718	N/A	N/A

Abbreviations: LVI: lymphovascular invasion; leptin-R: leptin receptor.

## Data Availability

Data are available upon reasonable request.

## Abbreviations

The following abbreviations are used in this manuscript:

AMPK	AMP-activated protein kinase
BMI	Body mass index
CI	Confidence interval
CSS	Cancer specific survival
H&E	Hematoxylin and eosin
HR	Hazard ratio
IMDC	International Metastatic Renal Cell Carcinoma Database Consortium
Leptin-R	Leptin receptor
LVI	Lymphovascular invasion
NAMPT	Nicotinamide phosphoribosyltransferase
NF-κB	Nuclear factor kappa B
OS	Overall survival
PFS	Progression-free survival
PPAR	Proliferator-activated receptor
RBP-4	Retinol binding protein 4
RFS	Recurrence-free survival
RCC	Renal cell carcinoma
TMA	Tissue microarray
TNM	Tumor, Node, Metastasis

## References

[1] Bray F., Laversanne M., Sung H., Ferlay J., Siegel R.L., Soerjomataram I., Jemal A. (2024). Global Cancer Statistics 2022: GLOBOCAN Estimates of Incidence and Mortality Worldwide for 36 Cancers in 185 Countries. CA Cancer J. Clin..

[2] Capitanio U., Bensalah K., Bex A., Boorjian S.A., Bray F., Coleman J., Gore J.L., Sun M., Wood C., Russo P. (2019). Epidemiology of Renal Cell Carcinoma. Eur. Urol..

[3] Wang F., Xu Y. (2014). Body Mass Index and Risk of Renal Cell Cancer: A Dose-response Meta-analysis of Published Cohort Studies. Int. J. Cancer.

[4] Vegiopoulos A., Rohm M., Herzig S. (2017). Adipose Tissue: Between the Extremes. EMBO J..

[5] Jung U., Choi M.-S. (2014). Obesity and Its Metabolic Complications: The Role of Adipokines and the Relationship between Obesity, Inflammation, Insulin Resistance, Dyslipidemia and Nonalcoholic Fatty Liver Disease. Int. J. Mol. Sci..

[6] Horiguchi A., Sumitomo M., Asakuma J., Asano T., Zheng R., Asano T., Nanus D.M., Hayakawa M. (2006). Increased Serum Leptin Levels and Over Expression of Leptin Receptors Are Associated with the Invasion and Progression of Renal Cell Carcinoma. J. Urol..

[7] de Martino M., Leitner C.V., Hofbauer S.L., Lucca I., Haitel A., Shariat S.F., Klatte T. (2016). Serum Adiponectin Predicts Cancer-Specific Survival of Patients with Renal Cell Carcinoma. Eur. Urol. Focus..

[8] Kallio J., Hämäläinen M., Luukkaala T., Moilanen E., Tammela T.L., Kellokumpu-Lehtinen P.-L. (2017). Resistin and Interleukin 6 as Predictive Factors for Recurrence and Long-Term Prognosis in Renal Cell Cancer. Urol. Oncol. Semin. Orig. Investig..

[9] Elkassem A.A., Allen B.C., Sharbidre K.G., Rais-Bahrami S., Smith A.D. (2021). Update on the Role of Imaging in Clinical Staging and Restaging of Renal Cell Carcinoma Based on the AJCC 8th Edition, From the *AJR* Special Series on Cancer Staging. Am. J. Roentgenol..

[10] Fuhrman S.A., Lasky L.C., Limas C. (1982). Prognostic Significance of Morphologic Parameters in Renal Cell Carcinoma. Am. J. Surg. Pathol..

[11] Paner G.P., Amin M.B., Alvarado-Cabrero I., Young A.N., Stricker H.J., Moch H., Lyles R.H. (2010). A Novel Tumor Grading Scheme for Chromophobe Renal Cell Carcinoma. Am. J. Surg. Pathol..

[12] Heng D.Y.C., Xie W., Regan M.M., Warren M.A., Golshayan A.R., Sahi C., Eigl B.J., Ruether J.D., Cheng T., North S. (2009). Prognostic Factors for Overall Survival in Patients with Metastatic Renal Cell Carcinoma Treated with Vascular Endothelial Growth Factor—Targeted Agents: Results From a Large, Multicenter Study. J. Clin. Oncol..

[13] Garofalo C., Surmacz E. (2006). Leptin and Cancer. J. Cell Physiol..

[14] Park H.-Y., Kwon H.M., Lim H.J., Hong B.K., Lee J.Y., Park B.E., Jang Y.S., Cho S.Y., Kim H.-S. (2001). Potential Role of Leptin in Angiogenesis: Leptin Induces Endothelial Cell Proliferation and Expression of Matrix Metalloproteinases In Vivo and In Vitro. Exp. Mol. Med..

[15] Dalamaga M., Diakopoulos K.N., Mantzoros C.S. (2012). The Role of Adiponectin in Cancer: A Review of Current Evidence. Endocr. Rev..

[16] Zhang Z., Du J., Xu Q., Li Y., Zhou S., Zhao Z., Mu Y., Zhao A.Z., Cao S.-M., Li F. (2022). Resistin Promotes Nasopharyngeal Carcinoma Metastasis through TLR4-Mediated Activation of P38 MAPK/NF-ΚB Signaling Pathway. Cancers.

[17] Frühbeck G., Jebb S.A., Prentice A.M. (1998). Leptin: Physiology and Pathophysiology. Clin. Physiol..

[18] Havel P.J. (2004). Update on Adipocyte Hormones. Diabetes.

[19] Tartaglia L.A., Dembski M., Weng X., Deng N., Culpepper J., Devos R., Richards G.J., Campfield L.A., Clark F.T., Deeds J. (1995). Identification and Expression Cloning of a Leptin Receptor, OB-R. Cell.

[20] Hoda M.R., Keely S.J., Bertelsen L.S., Junger W.G., Dharmasena D., Barrett K.E. (2007). Leptin Acts as a Mitogenic and Antiapoptotic Factor for Colonic Cancer Cells. Br. J. Surg..

[21] Babaei A., Zarkesh-Esfahani S.H., Bahrami E., Ross R.J. (2011). Restricted Leptin Antagonism as a Therapeutic Approach to Treatment of Autoimmune Diseases. Hormones.

[22] Choi S.H., Chun S.Y., Kim T.-H., Kwon T.G. (2016). Identifying the Emerging Role of Adipokine as a Diagnostic and Prognostic Biomarker of Renal Cell Carcinoma. Urol. Oncol. Semin. Orig. Investig..

[23] Zhu H., Li W., Mao S., Wang L. (2018). Association between Leptin Level and Renal Cell Carcinoma Susceptibility and Progression. J. Cancer Res. Ther..

[24] Dimou N.L., Papadimitriou N., Mariosa D., Johansson M., Brennan P., Peters U., Chanock S.J., Purdue M., Bishop D.T., Gago-Dominquez M. (2021). Circulating Adipokine Concentrations and Risk of Five Obesity-related Cancers: A Mendelian Randomization Study. Int. J. Cancer.

[25] Perumal K., Mun K.S., Yap N.Y., Razack A.H.A., Gobe G.C., Ong T.A., Kuppusamy S., Rajandram R. (2020). A Study on the Immunohistochemical Expressions of Leptin and Leptin Receptor in Clear Cell Renal Cell Carcinoma. Biomed. Res. Int..

[26] Lee Y.-C., Wu W.-J., Lin H.-H., Li W.-M., Huang C.-N., Hsu W.-C., Chang L.-L., Li C.-C., Yeh H.-C., Li C.-F. (2017). Prognostic Value of Leptin Receptor Overexpression in Upper Tract Urothelial Carcinomas in Taiwan. Clin. Genitourin. Cancer.

[27] Horiguchi A., Sumitomo M., Asakuma J., Asano T., Zheng R., Asano T., Nanus D.M., Hayakawa M. (2006). Leptin Promotes Invasiveness of Murine Renal Cancer Cells Via Extracellular Signal-Regulated Kinases and Rho Dependent Pathway. J. Urol..

[28] Jeong Y.-J., Bong J.-G., Park S.-H., Choi J.-H., Oh H.-K. (2011). Expression of Leptin, Leptin Receptor, Adiponectin, and Adiponectin Receptor in Ductal Carcinoma In Situ and Invasive Breast Cancer. J. Breast Cancer.

[29] Ng K.L., Del Vecchio S.J., Samaratunga H., Morais C., Rajandram R., Vesey D.A., Wood S.T., Gobe G.C. (2018). Leptin and Its Receptor: Can They Help to Differentiate Chromophobe Renal Cell Carcinoma from Renal Oncocytoma?. Pathology.

[30] Uddin S., Bu R., Ahmed M., Abubaker J., Al-Dayel F., Bavi P., Al-Kuraya K.S. (2009). Overexpression of Leptin Receptor Predicts an Unfavorable Outcome in Middle Eastern Ovarian Cancer. Mol. Cancer.

[31] Wilson K.M., Cho E. (2016). Obesity and Kidney Cancer. Obesity and Cancer.

[32] Arita Y., Kihara S., Ouchi N., Takahashi M., Maeda K., Miyagawa J., Hotta K., Shimomura I., Nakamura T., Miyaoka K. (1999). Paradoxical Decrease of an Adipose-Specific Protein, Adiponectin, in Obesity. Biochem. Biophys. Res. Commun..

[33] Wei E.K., Giovannucci E., Fuchs C.S., Willett W.C., Mantzoros C.S. (2005). Low Plasma Adiponectin Levels and Risk of Colorectal Cancer in Men: A Prospective Study. JNCI J. Natl. Cancer Inst..

[34] Spyridopoulos T.N., Petridou E.T., Skalkidou A., Dessypris N., Chrousos G.P., Mantzoros C.S., The Obesity and Cancer Oncology Group (2007). Low Adiponectin Levels Are Associated with Renal Cell Carcinoma: A Case-control Study. Int. J. Cancer.

[35] Michalakis K., Williams C.J., Mitsiades N., Blakeman J., Balafouta-Tselenis S., Giannopoulos A., Mantzoros C.S. (2007). Serum Adiponectin Concentrations and Tissue Expression of Adiponectin Receptors Are Reduced in Patients with Prostate Cancer: A Case Control Study. Cancer Epidemiol. Biomark. Prev..

[36] Curat C.A., Wegner V., Sengenès C., Miranville A., Tonus C., Busse R., Bouloumié A. (2006). Macrophages in Human Visceral Adipose Tissue: Increased Accumulation in Obesity and a Source of Resistin and Visfatin. Diabetologia.

[37] Hivert M.-F., Sullivan L.M., Fox C.S., Nathan D.M., D’Agostino R.B., Wilson P.W.F., Meigs J.B. (2008). Associations of Adiponectin, Resistin, and Tumor Necrosis Factor-α with Insulin Resistance. J. Clin. Endocrinol. Metab..

[38] Liao L.M., Weinstein S.J., Pollak M., Li Z., Virtamo J., Albanes D., Chow W.-H., Purdue M.P. (2013). Prediagnostic Circulating Adipokine Concentrations and Risk of Renal Cell Carcinoma in Male Smokers. Carcinogenesis.

[39] Zhang H.-P., Zou J., Xu Z.-Q., Ruan J., Yang S.-D., Yin Y., Mu H.-J. (2017). Association of Leptin, Visfatin, Apelin, Resistin and Adiponectin with Clear Cell Renal Cell Carcinoma. Oncol. Lett..

